# Ponatinib as a Valid Alternative Strategy in Patients with Blast Crisis-Chronic Myeloid Leukemia Not Eligible for Allogeneic Stem Cells Transplantation and/or Conventional Chemotherapy: Report of a Case

**DOI:** 10.1155/2017/6167345

**Published:** 2017-08-14

**Authors:** Cristina Bucelli, Daniele Cattaneo, Valeria Ferla, Manuela Zappa, Caterina de Benedittis, Simona Soverini, Alessandra Iurlo

**Affiliations:** ^1^Hematology Division, IRCCS Ca' Granda-Maggiore Policlinico Hospital Foundation and University of Milan, Milan, Italy; ^2^Department of Experimental, Diagnostic and Specialty Medicine, Institute of Hematology “L. and A. Seragnoli,” University of Bologna, Bologna, Italy

## Abstract

Currently, imatinib and dasatinib are the only tyrosine-kinase inhibitors approved in the US and Europe for the treatment of blast crisis of chronic myeloid leukemia (BC-CML) at diagnosis, while ponatinib is the only inhibitor used in patients bearing T315I mutation. Here we report the case of a 61-year-old man diagnosed with B-cell lymphoid BC-CML, initially treated with imatinib 800 mg day and then with dasatinib 140 mg day because of intolerance. A complete cytogenetic response (CCyR) was achieved at three months; however, three months later a relapse was observed, and the T315I mutation was detected. Ponatinib 45 mg once daily was then started together with a short course of chemotherapy. Bone marrow evaluation after six months of therapy showed the regaining of CCyR, together with the achievement of a deep molecular response. However, one year from ponatinib start the patient experienced a new disease relapse; he was effectively treated with ponatinib and chemotherapy once again, but in the meanwhile an ischemic stroke was detected. This case report confirms the high efficacy of ponatinib monotherapy in BC-CML patients, representing a valid option for non-allogeneic stem cells transplantation eligible cases and the only one available for those carrying the T315I mutation.

## 1. Introduction

Chronic myeloid leukemia (CML) prognosis changed dramatically with the development of tyrosine-kinase inhibitors (TKIs) that potently interfere with the interaction between the* BCR-ABL1* protein and adenosine triphosphate (ATP), thus blocking proliferation of the malignant cellular clone [1]. This targeted approach has markedly increased survival of CML patients and greatly reduced the frequency of blast crisis (BC) compared to the pre-TKI era. In fact, the incidence of BC now ranges between 0.7 and 4.5%, showing the highest incidence in the first year after diagnosis and then decreasing [2, 3].

A percentage of patients between 0.9 and 6.7% are already in BC at diagnosis [2–5]. More recently, in the EUTOS population-based registry, the incidence of BC at diagnosis was found to be about 2.2% [3]. Median survival ranges between 6 and 11.8 months [6].

According to the European LeukemiaNet (ELN) recommendations, BC is defined using the threshold blast count in the peripheral blood or bone marrow at 30% [7].

Up to 80% of all BC patients show additional chromosomal aberrations (ACA) [8], and at least 77% carry mutations in the* ABL1* domain. Therefore, their mutational status should also be evaluated, since it is associated with different prognoses, for example, T315I mutation-positive cases compared to others [9–11].

At diagnosis, treatment mainly depends on the type of BC (myeloid versus lymphoid), mutational status, and previous therapies. Treatment options may include TKIs, conventional chemotherapy, allogeneic stem cells transplantation (HSCT), and/or other treatment modalities. To date, imatinib and dasatinib are the only TKIs approved in the US and Europe for treatment of BC at diagnosis, while ponatinib is the only one which can be used also in patients harboring T315I mutation [12–16].

Here we report the case of a 61-year-old man diagnosed with B-cell lymphoid BC-CML initially treated with imatinib, which was soon suspended because of intolerance, followed by dasatinib. Then, he acquired a T315I mutation but achieved a deep molecular response (MR4) on ponatinib therapy. Furthermore, ponatinib monotherapy was able to maintain this patient in CML-chronic phase for about one year before he died because of an infective complication.

## 2. Case Presentation

**Figure 1:**
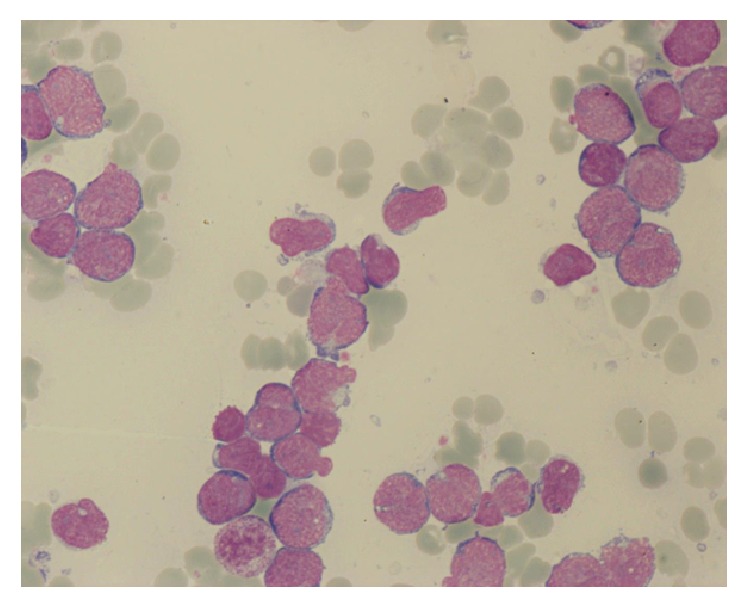
Bone marrow aspirate morphology at diagnosis, showing atypical granular lymphoblasts.

In January 2015, a 61-year-old man was referred to our hospital because of asthenia, diffuse bone pain, fever, and cutaneous hemorrhagic diathesis. Laboratory tests showed a mild anemia (hemoglobin, 12 g/dL), leukocytosis (white blood cells count, 22 × 10^9^/L, with neutrophils 57%; lymphocytes 11%; monocytes 2%; eosinophils 6%; basophils 1%; myelocytes 6%; metamyelocytes 4%; blast cells 13%), and severe thrombocytopenia (platelet count, 4 × 10^9^/L). The patient was obese and suffered from chronic bronchitis; in addition, he was an active smoker (about 40 cigarettes per day) and an alcohol abuser, with three previous episodes of suicide attempt. Bone marrow morphological analysis showed an atypical granular lymphoblastic infiltration of 70% (Figure 1), which was confirmed by flow cytometry immunophenotyping (high positivity for CD10/CD19/CD34/CD99/CD79a/TdT; weak positivity for CD33 (60%)/CD38 (80%); negativity for CD3/CD13/CD14/CD20/CD117/MPO). Cytogenetic study of 20 metaphases revealed a typical 46, XY, t(9;22)(q34;q11.2) translocation, and qualitative polymerase chain reaction (PCR) demonstrated the presence of the* BCR-ABL1* fusion transcript, with a typical e13a2 configuration. Baseline assessment of* BCR-ABL1* mutational status with Sanger sequencing was negative, datum which was also retrospectively confirmed by ultradeep sequencing. Therefore, a diagnosis of B-cell lymphoid BC of CML was made. As a matched sibling donor was not available and the patient categorically refused HSCT, we decided not to start a search for a matched unrelated donor. Because of a previous exposition to hepatitis B virus, a prophylactic treatment with lamivudine was initiated.

The patient was initially treated with imatinib 800 mg day, which was suspended approximately after one month because of intolerance (grade III crippling cramps, periorbital edema, and conjunctival hemorrhage), and then he started dasatinib 140 mg day. At the same time, methylprednisolone was administered at a full dose and then decreased for the appearance of diabetes which required treatment with oral hypoglycemic drugs and then also with insulin.

Hematological response was obtained after one month, and at three months bone marrow evaluation showed a complete cytogenetic response (CCyR), together with a* BCR-ABL1* transcript level of 0.2% according to the International Scale (IS).

Nevertheless, three months later a BC-CML relapse was observed, with a* BCR-ABL1* transcript level of 89.5% according to IS, and a new evaluation of* BCR-ABL1* mutational status revealed the presence of T315I mutation. Cytological and flow cytometric analyses of cerebrospinal fluid showed no leukemic cells.

As suggested by ELN 2013 recommendations, ponatinib 45 mg once daily was started together with chemotherapy (vincristine intravenously every seven days for three cycles and oral prednisone). Due to patient's comorbidities, an antiplatelet therapy with ASA 100 mg day was coprescribed in order to reduce the risk of arterial thrombotic events.

After three months of ponatinib therapy, bone marrow evaluation showed the regaining of a CCyR and a* BCR-ABL1* transcript of less than 10% according to IS.

The drug was fairly well tolerated, even though after two months severe upper abdominal burning pain irradiating to the back appeared. Blood analysis showed an increase in lipase and amylase levels (grade III pancreatic toxicity), and an abdomen CT scan showed a mild pancreatitis. It was then decided to maintain ponatinib at the same dosage and to strictly monitor the patient, achieving then a spontaneous resolution of all symptoms.

**Figure 2:**
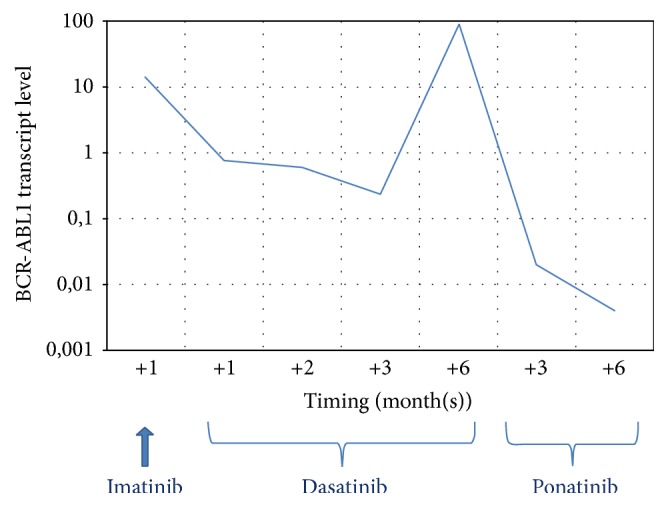
*BCR-ABL1* transcript level during TKIs therapy.

Bone marrow evaluation after six months of therapy confirmed CCyR, together with the achievement of a deep molecular response, with a* BCR-ABL1* transcript level of 0.004% according to IS (MR4) (Figure 2).

Nevertheless, after about one year from ponatinib start, the patient experienced disease relapse: bone marrow morphological analysis showed an atypical granular lymphoblastic infiltration of 55%, which was confirmed by flow cytometry immunophenotyping (high positivity for CD10/CD19/CD79a/CD99/TdT; weak positivity for CD33 (30%)/CD38 (50%); negativity for CD20/CD34). In addition, a new cytogenetic abnormality was detected, his karyotype being the following: 46,XY,t(9;22)(q34,q11.2) [3]/47,XY,t(9;22)(q34;q11.2), +der(1;19)(p11;q11) [4].

Due to the good response to the previous chemotherapy, a new course of intravenous vincristine and oral prednisone was started. However, after the second cycle of chemotherapy, the patient showed dysarthria and weakness of the right leg; therefore he was hospitalized to clarify the origin of these new neurological symptoms. With the hypothesis of a central nervous system (CNS) localisation of lymphoid BC-CML, a new cytological and flow cytometric analysis of cerebrospinal fluid was performed but it showed no leukemic cells; on the contrary, a CNS CT scan demonstrated an ischemic stroke with numerous recent lesions in the periventricular area and within the frontal-parietal cortex of the left hemisphere. Accordingly, due to the vascular etiology of the ischemic event, anticoagulant therapy with low molecular weight heparin (LMWH) was added to ASA, and statins and antihypertensive therapies were started. Meanwhile, ponatinib and intravenous vincristine were regularly administered, leading to the rapid achievement of a complete normalisation of blood cells count with no need of red blood cells or platelets transfusion.

Approximately after two months from hospitalization, the patient developed a septic shock due to a pulmonary infection not responsive to empiric antibiotic therapy and died.

## 3. Discussion

Even if TKIs have completely changed the outcome and survival of CML patients, treatment of advanced phases still remains a clinical challenge, particularly for patients not eligible to HSCT, which remains to date the only potentially curative strategy for eligible patients.

In the case reported here, the patient was not eligible to HSCT due to his comorbidities correlated with a Sorror score of 8, identifying a 2-year non-relapse mortality of 41% and a 2-year overall survival of 34% [17].

In addition, due to the complexity of psychological characteristics of the patient who refused not only HSCT but also hospitalization, he was initially treated with TKI monotherapy. As reported in the literature, hematologic and cytogenetic responses with single agent TKIs are achieved in about 50 and 12% of the cases, respectively, with the 12-month overall response ranging between 25 and 49% and the median survival between 6 and 11.8 months [6]. Outcome of lymphoid BC-CML treated with dasatinib varies between 39 and 80% of hematologic remission and from 40 to 90% of any cytogenetic response [6, 18, 19]. Concerning molecular response, no data have been reported in the literature so far. Of note, in a retrospective analysis aimed at characterizing mutation development in patients with newly diagnosed CML treated with imatinib or dasatinib frontline, a narrower spectrum of mutations in dasatinib-treated patients was found, with the occurrence of T315I mutation in approximately 17% of cases [20]. Indeed, our patient experienced disease relapse within six months of dasatinib treatment with the occurrence of T315I mutation.

Since the patient was in BC at the time of diagnosis, he was investigated for* BCR-ABL1* mutational status by Sanger sequencing and found negative. However, Soverini et al. [21] and Baer et al. [22] reported that the T315I mutation can be identified by means of ultradeep sequencing approximately three months before the Sanger sequencing detection threshold is reached. Therefore, the same specimen was retrospectively analyzed by ultradeep sequencing, but despite a sequencing depth of 7614 reads (theoretically enabling a sensitivity of approximately 0.03%) no T315I mutation was detected. Interestingly, in 4% of all alleles, a 35 bp insertion, already reported in the literature as “35INS,” was identified. The contribution of this insertion to TKI resistance is still a matter of debate. However, a recent functional* in vitro* study has indicated that the* BCR-ABL1* ^35INS^ isoform is not a functional kinase, thus it should not have any role in sustaining the disease and triggering drug resistance [23].

Due to the low incidence of BC-CML, studies on the combination of TKIs with chemotherapy are rather scanty and usually with a limited number of enrolled patients. Considering that patients with lymphoid BC can be treated with acute lymphoblastic leukemia- (ALL-) type induction regimens, with our patient being not eligible to HSCT, we decided to administer three courses of vincristine and high dose corticosteroids in association with ponatinib. Since only ponatinib shows activity also against T315I mutation, it was administered at full dose in our patient, despite its potential side effect profile. As reported in phase 2 PACE study of ponatinib in Ph+ leukemias, the median time to a major hematologic response in BC-CML patients was 4.1 weeks (range, 1.7 to 16.1), and the estimated rate of a sustained response at 12 months was 42%. Overall survival was estimated to be 29% at 12 months (median, 7 months), but no data were reported about achievement of major molecular response (MMR) [16].

Response to treatment is the most important prognostic factor for survival in BC-CML patients [24, 25]. In particular, best results are obtained in patients returning to chronic phase especially if cytogenetic or molecular responses are achieved.

Concerning ponatinib safety profile, arterial thrombotic events are reported in about 22% of all treated patients, showing a linear correlation with the drug dosage [16]. It has to be considered that our patient bears different cardiovascular risk factors, including hypertension, hypercholesterolemia, steroid-induced diabetes, alcohol abuse, smoking habit, and obesity; however, ponatinib at a dosage of 45 mg day represented the only possible therapeutic option for such a complex subject.

Our data confirm the high efficacy of ponatinib in BC-CML patients and the potent activity of this drug also in achieving molecular response in such patients and maintaining the chronic phase of the disease for almost one year. In addition, another significant aspect in this case is represented by the good quality of life for the patient, who remained an outpatient for the entire treatment duration.

In conclusion, ponatinib monotherapy or in association with chemotherapy is a valid treatment option for non-HSCT eligible BC-CML patients and the only one available for those carrying the T315I mutation, which can develop during dasatinib therapy frequently.
